# Fickian yet non-Gaussian diffusion of a quasi-2D colloidal system in an optical speckle field: experiment and simulations

**DOI:** 10.1038/s41598-023-34433-z

**Published:** 2023-05-06

**Authors:** Antonio Ciarlo, Raffaele Pastore, Francesco Greco, Antonio Sasso, Giuseppe Pesce

**Affiliations:** 1 Department of Physics E. Pancini, University of Naples Federico II, Complesso Universitario Monte Sant’Angelo, Via Cintia, 80126 Naples, Italy; 2 Department of Chemical, Materials and Production Engineering, University of Naples Federico II, P.le Tecchio 80, 80125 Naples, Italy

**Keywords:** Optical techniques, Statistical physics, thermodynamics and nonlinear dynamics

## Abstract

We investigate a quasi-2D suspension of Brownian particles in an optical speckle field produced by holographic manipulation of a laser wavefront. This system was developed to study, in a systematic and controllable way, a distinctive instance of diffusion, called Fickian yet Non Gaussian diffusion (FnGD), observed, during the last decade, for colloidal particles in a variety of complex and biological fluids. Our setup generates an optical speckle field that behaves like a disordered set of optical traps. First, we describe the experimental setup and the dynamics of the particles, focusing on mean square displacements, displacement distributions and kurtosis. Then, we present Brownian Dynamics simulations of point-like particles in a complex energy landscape, mimicking that generated by the optical speckle field. We show that our simulations can capture the salient features of the experimental results, including the emergence of FnGD, also covering times longer than the ones so far achieved in experiments. Some deviations are observed at long time only, with the Gaussian restoring being slower in simulations than in experiments. Overall, the introduced numerical model might be exploited to guide the design of upcoming experiments targeted, for example, to fully monitor the recovery of Gaussianity.

## Introduction

The phenomenon of thermally driven diffusion, which controls fundamental mechanisms in many areas of science^1–3^, shows aspects not yet fully understood. From the pioneering works of Einstein^4^, it emerged that Brownian diffusion has two fundamental characteristics: the Mean Squared Displacement (MSD) increases linearly in time, and the Displacement Distribution (DD) is Gaussian. These relationships have been universally accepted one-to-one for a long time, in the absence of experimental evidence about their decoupling. Indeed, the well known anomalous diffusion is neither Gaussian nor Fickian^5–7^.

In 2009, however, Wang et al.^8^ observed a novel type of diffusion, in which Fickianity is not accompanied by Gaussianity (therefore termed Fickian yet not Gaussian diffusion, FnGD, or Brownian non-Gaussian diffusion), studying the motion of colloidal particles in F-actin filament networks or along micro-tubules. While ref.^8^ reports the first sharp claim of FNGD, interesting clues of the possible existence of this phenomenon were already inferable from earlier works, e.g. from experiments on glassy colloidal suspensions^9^. After ref.^8^, on the other hand, FnGD has been explicitly reported for a variety of systems, such as hard-sphere colloidal suspensions^10^, supercooled liquids close to the glass transition^11,12^, bacterial cytoplasms^13^, a variety of other soft matter systems^14–20^, and also in markets price dynamics^21^. FnGD, therefore, has been the subject of theoretical studies^22–25^ drawing on various modeling approaches, but the phenomenon is still not fully explained.

From an experimental perspective, identifying an appropriate physical system is crucial to carry out a systematic study of FnGD with fine control of its key parameters. To this aim, in recent experiments^26,27^, we exploited a quasi-2D model-system of Brownian microparticles embedded in an optical speckle field generated by a holographic manipulation of the wavefront of a laser beam. The optical speckle field behaves like a random optical potential in which the microparticles experience weak optical forces modifying their trajectories.

Brownian motion in random energy potential landscapes has been extensively studied in the literature, from an experimental and theoretical point of view, or by numerical simulations^28–32^. These studies focused, for example, on diffusion in disordered media^5,33^, on the diffusion of particles in non-homogeneous media such as the dynamics of single molecules through pores^34^ and inside cells^35,36^, on the study of the dynamics of impurities in conductors^37,38^, and on the study of diffusion in *random harmonic potentials*^39,40^. Thanks to the development of optical holographic techniques, 1-D and 2-D random optical potentials generated by light raised a great deal of interest^41,42^. Nevertheless, random optical potentials have never been used to study FnGD, as proposed in our work.

In this article, we push forward the experimental study of FnGD in this system, complementing experimental results with numerical simulations.

## Optical random-energy landscapes generation: an on-demand environment for FnGD

### Experimental setup

**Figure 1:**
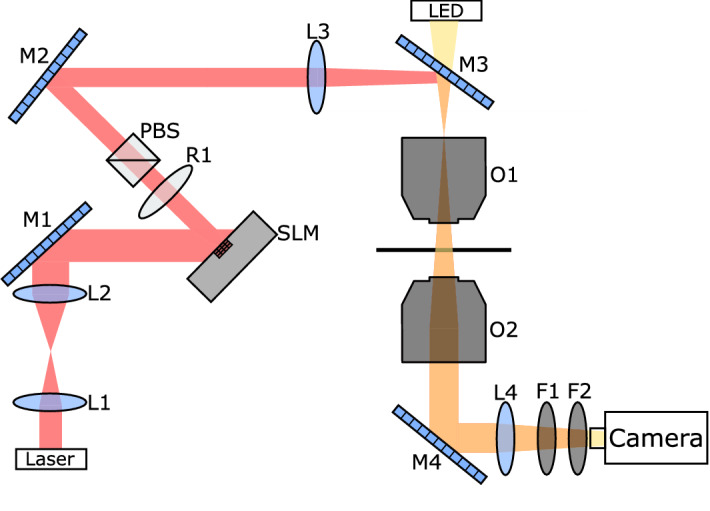
Schematic representation of experimental setup. In figure M1, M2, M3 and M4 are dichroic mirrors, O1 and O2 objectives, L1, L2, L3 and L4 lenses, R1 a polarizer, PBS a polarized beam splitter, and F1 and F2 infrared filters.

As recently demonstrated^26,27^, a disordered optical landscape represents an ideal system to create a heterogeneous environment for Brownian particles, where the FnGD regime can be studied in detail. In fact, several parameters can be finely tuned, like the density and the roughness of the optical potential landscape.

Our setup is schematically shown in Fig. 1. A TEM$_{00}$ laser beam ($\lambda =1064\,$nm) (IPG PYL-10M), is enlarged with a couple of lenses (L1 and L2) in telescopic configuration to uniformly illuminate the SLM sensor (LCOS-SLM X10468-3) which modifies the wavefront phase of the laser field. The objective O1 produces in its focal plane the Fourier transform of the modified wavefront and, hence, generates the optical speckle field. The grain size of the speckle can be varied acting on the SLM mask, while the speckle optical intensity is changed acting on the laser power with the polarizer beam splitter PBS. The colloidal sample, placed in the focal plane of O1, is illuminated with a LED thanks to the dichroic mirror M3. The transmitted light through the sample is collected by the objective O2 and focused onto the calibrated CCD camera. Infrared laser radiation is properly filtered by filters F1 and F2 to allow a simultaneous image of the colloidal pattern and of the optical speckle field^43^. CCD camera images were calibrated with a micrometric ruler giving a conversion factor of 0.203 μm/pixel.

### Characterization of the optical speckle field

**Figure 2:**
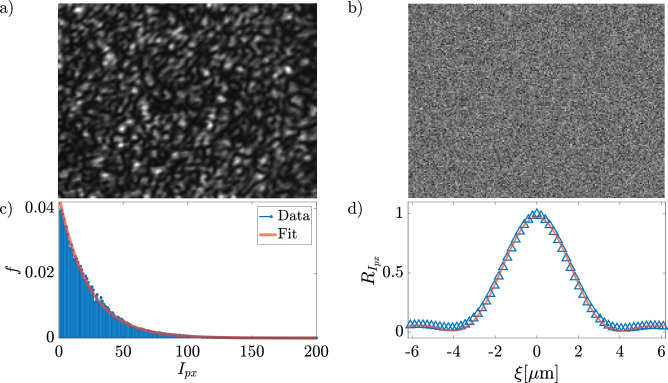
(**a**) a typical image of the optical speckle field with a power of 0.67 W acquired in the sample plane, and (**b**) the corresponding SLM mask. (**c**) histogram of pixel intensity and (**d**) the radial average of the auto-correlation function of the optical speckle field in (**a**). The solid lines are the fitting curves obtained with Eqs. (1) and (2).

When the phase of a monochromatic optical field is randomly modified point-by-point, the far field forms a pattern of disordered optical spots (grains), like in Fig. 2a. An optical speckle field is characterized by two principal statistical properties^44^:

- the intensity distribution obeys to a negative exponential statistics:
  1 $$\begin{aligned} P(I)\propto \exp \left[ -\frac{I}{E[I]}\right] \end{aligned}$$
  where $E[\cdot ]$ represents the expected value and *I* is the intensity of the optical field;
- the auto-correlation intensity function, in our case of a uniform and square scattering spot, is given by:
  2 $$\begin{aligned} R_I(\Delta x, \Delta y)=E[I]^2\left[ 1+\text {sinc}^2\left( \frac{L\Delta x}{\lambda z}\right) \text {sinc}^2\left( \frac{L\Delta y}{\lambda z}\right) \right] \end{aligned}$$
  where *L* is the linear dimension of a squared scattering area, $\lambda$ is the laser wavelength and *z* is the distance between the scattering area and the observation plane. This function can be approximated by a Gaussian function, the mean grain size being about 3 times the standard deviation.

To produce an optical speckle field, we pilot the SLM with a simple image file (called mask, Fig. 2b) that changes the laser wave-front phase point-by-point. A typical optical speckle field so generated is shown in Fig. 2a.

**Figure 3:**
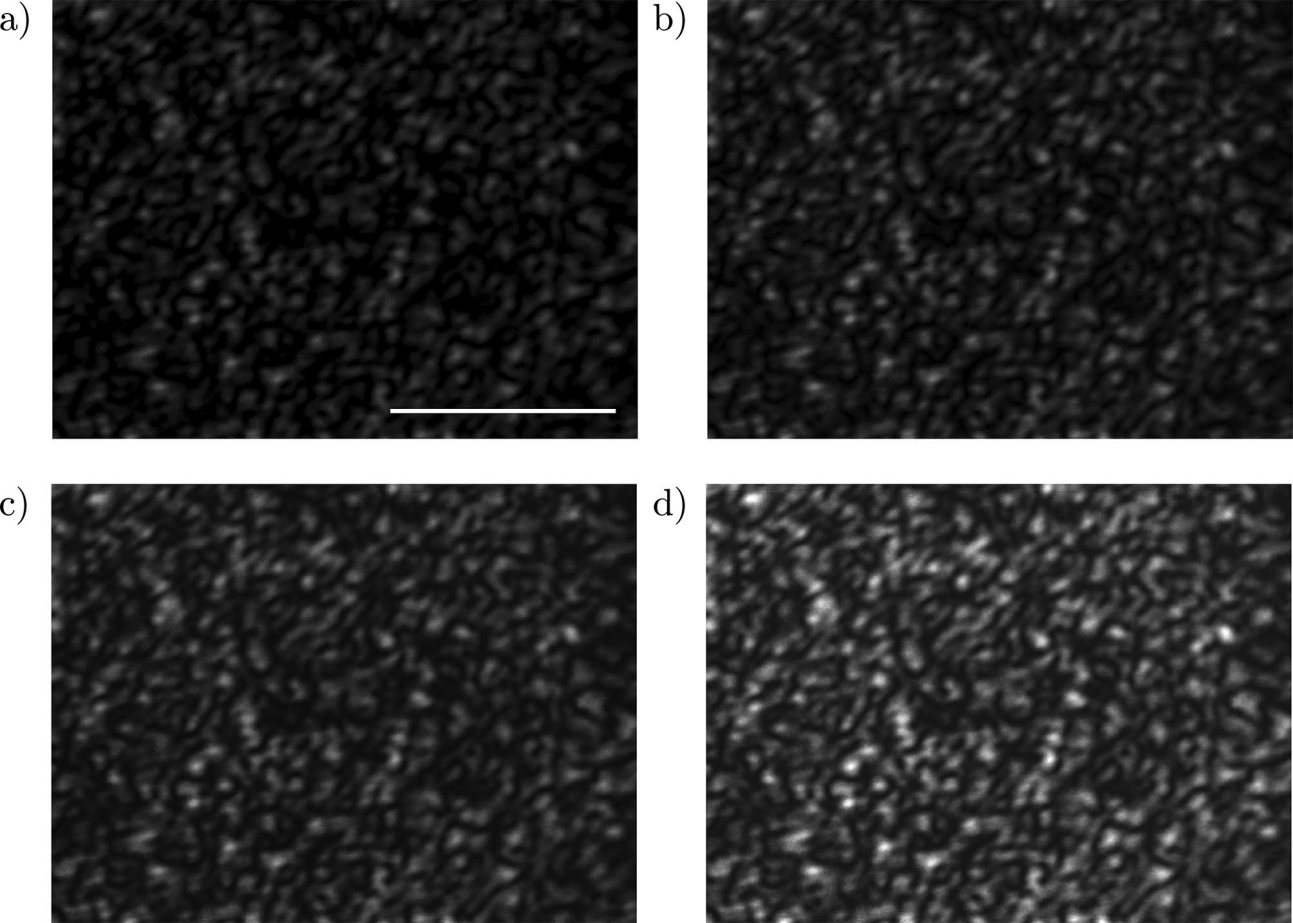
Optical speckle fields for different laser power on the sample (**a**) 0.15 W, (**b**) 0.43 W, (**c**) 0.61 W, and (**d**) 0.67 W. The white line in panel (**a**) has a length of 50 μm.

Firstly, we checked the statistical properties of the generated optical field. We found that the intensity distribution and the auto-correlation function are well fitted by (1) and (2), respectively, as shown in Fig. 2c and d. From the fit, we obtained the mean grain size $\Delta _g\sim 4\,\mu$m as the distance between the central maximum and the first minimum^44^. In our case, $\Delta _g$ is slightly larger than the diameter of the silica spheres used in the experiment.

With our setup we can change three of the several parameters that may affect FnGD: (i) the size of the microparticles, (ii) the size of the speckle grains, and (iii) the optical intensity of the speckle field. In this experiment, we kept the first two parameters fixed and varied the laser power P on the sample, studying the case of P = 0.43, 0.61, and 0.67 W. In order to check that the laser power does not affect the optical speckle field, we have recorded the images of several optical speckle fields produced with the same mask but at different laser powers (Fig. 3) keeping fixed the camera settings (exposure time, gain, frame-per-second). We did not observe any difference in the intensity exponential distribution and auto-correlation length, but only an increase in the intensity as clearly visible in Fig. 3.

### Sample preparation and tracking

**Figure 4:**
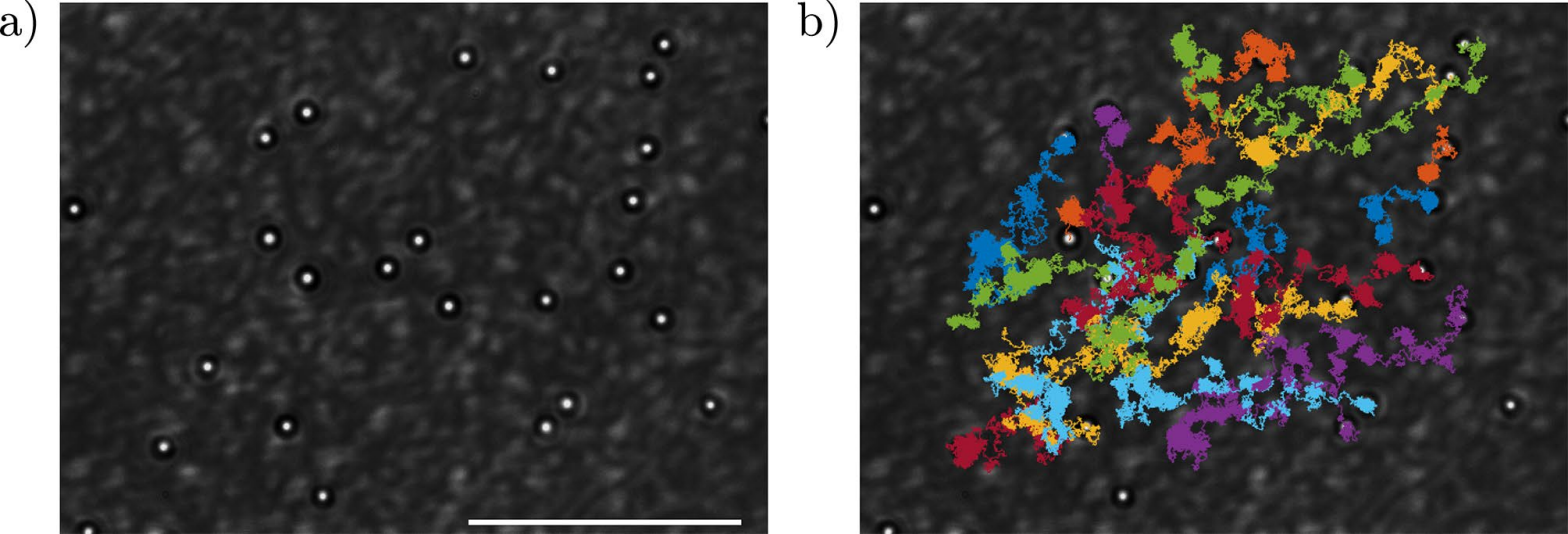
Sample image acquired without one of the two IR filters (**a**) and including the recorded particle trajectories (**b**). The scale bar is 50 μm.

In this experiment, we used a sample cell consisting of a channel made of two glass slides (1 mm and 150 μm thick) separated by 100 μm thick spacers. Silica microparticles (diameter $d=2.31\,\upmu$m) were diluted in water and 0.01% (V/V) surfactant (Triton X-100) to avoid particles sticking to the glass surface. The solution was injected into the channel which was then sealed with UV glue. It is important to point out that microparticles move essentially on the sample cell bottom plane (quasi 2-D diffusion) because silica is denser than water and the scattering force pushes microparticles in the same direction of gravity force. So, in our configuration, the optical scattering force does not affect the motion in the x-y plane that is influenced only by the gradient force. In addition, a special care has been taken to avoid drift motion due to small inclinations of the sample cell. The microparticles have a diameter slightly smaller than the average grain size, in a ratio $d/\Delta _g \sim 0.6$, Fig. 4a.

A Prosilica GE680 CCD camera acquired video files at 10 fps for 1 h (36,000 frames). Particle centroids were tracked using a well-known algorithm^45^ (see Fig. 4b). In our analysis, we considered trajectories that remain in the field-of-view of our microscope for the whole acquisition time. This is the reason why some of the microparticles shown in Fig. 4b are displayed without their corresponding trajectory.

The whole setup was enclosed in a box to avoid thermal drift and the temperature was continuously monitored. During the experiments, the temperature remained stable at the value T = 24.0 ± 0.5 °C.

### Characterization of the optical potential landscape

The analysis of the trajectories of the microparticles allows us to reconstruct the potential landscape felt by the particle. The potential *U*(*x*, *y*) is reconstructed from the distributions of the positions visited by the particles *p*(*x*, *y*) according to Boltzmann’s formula^43,46,47^:

3 $$\begin{aligned} p(x,y) \propto e^{-\frac{U(x,y)}{k_BT}} \Longrightarrow U(x,y) = -k_BT \ln [p(x,y)] +c_0 \end{aligned}$$

where $k_B$ is the Boltzmann constant, *T* is the temperature of the sample, and $c_0$ is an arbitrary constant chosen to give a potential equal to zero in the case of particles that diffuses freely, that is when the optical speckle field is off.

The reconstruction of the landscape of the optical potential is not trivial due to some experimental limitations. Indeed, the number of trajectories, although large, is not sufficient to cover the whole observed optical speckle field, e.g. some pixels of the speckle image are never visited by a particle during the observation time. This implies also that the statistics of occupational frequencies at certain points might be inadequate.

To face this issue, we used the following procedure: (i) we counted the number of visits in each specific location for every trajectory; (ii) we summed the number of counts; (iii) we divided it by the number of the only trajectories that pass across that location. Finally, the locations with a number of visits less than 5 counts were discarded from the reconstruction of the optical potential.

**Figure 5:**
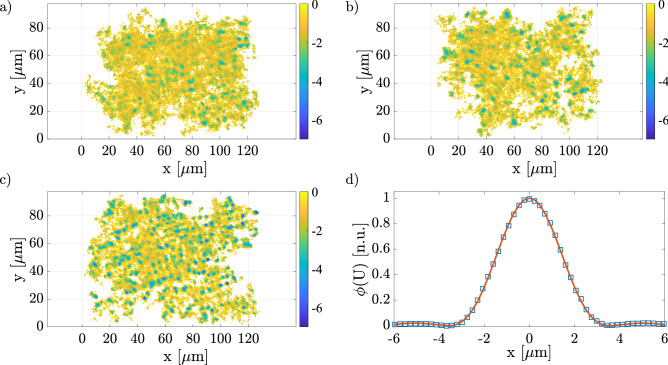
(**a**–**c**) Optical potential landscapes obtained from the trajectories recorded at laser power P = 0.43 W, 0.61 W, and 0.67 W respectively. The colorbars are in unit of $k_BT$; (**d**) auto-correlation function radial mean of the potential field at power 0.67 W.

The results for the three different laser powers are shown in Fig. 5. Obviously, as the laser power increases, the potential wells become deeper and the particles spend most of their time inside them. We also evaluated the auto-correlation function of the potential, Fig. 5d. Following the same procedure used for the auto-correlation function of the optical speckle field, we fitted the data to Eq. (2) finding a value for the correlation length of the potential of $\Delta _P\sim 3.5\ \upmu$m, comparable with the mean grain size of the optical speckle field $\Delta _g\sim 4\,\upmu$m.

## Analysis of the diffusion process

### Average mean squared displacement

**Figure 6:**
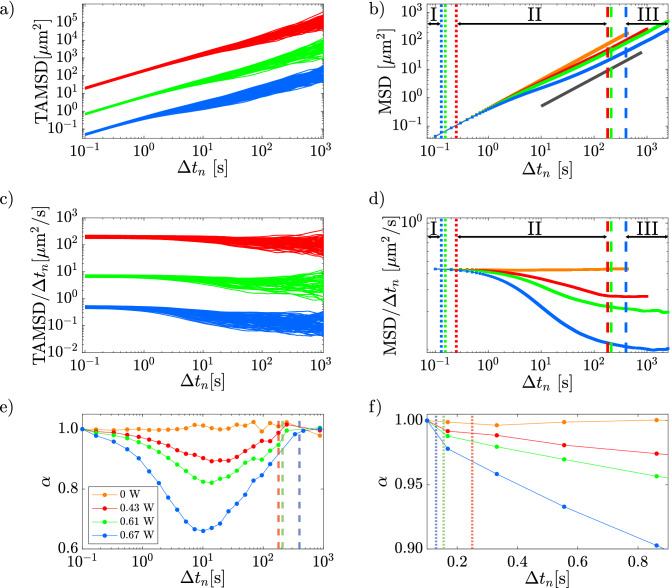
(**a**) TAMSD for laser power 0.43 W (red lines), 0.61 W (green lines) and 0.67 W (blue lines); (**b**) MSD versus $\Delta t_n$, where the gray solid line with slope 1 is a guide for the eye; (**c**) TAMSD$/\Delta t_n$ and (**d**) MSD$/\Delta t_n$ as a function of $\Delta t_n$; (**e**) $\alpha$ versus $\Delta t_n$; (**f**) zoom of  the panel (**e**) to show the transition between region I and II. In all panels, the dotted lines indicate $\tau _{I/II}$, and the dashed lines $\tau _{II/III}$.  Data in panel (**a**) and (**c**) have been shifted for clarity.

For every single particle-trajectory, we calculated the Time Average MSD (TAMSD) as:

4 $$\begin{aligned} \text {TAMSD}(\Delta t_n)=\frac{1}{N-n}\sum _{\xi =x,y}\sum _{k=1}^{N-n}\left[ \xi (t_{k}+\Delta t_n)-\xi (t_{k})\right] ^2 \end{aligned}$$

where *N* is the number of video frames (36,000 for our work), $n=1, \ldots ,N-1$, $\xi (t_k)$ is one of the two coordinates $(x(t_k),y(t_k))$ of the sphere center at time $t_k=k\tau$, $\Delta t_n=n\tau$ is the time increment (lag time). In our case $\tau =0.1\,$s. The typical behavior of the TAMSDs for a subset of about 35 trajectories is shown in Fig. 6a.

Averaging all the TAMSD for a fixed value of the optical power, we obtained the “traditional” MSD, also known as Time and Ensemble average MSD (TEAMSD). The MSD provides a direct way to represent the global behavior of the colloidal microparticles. In our experiments, averages for each laser power are performed over hundreds of trajectories. The MSDs obtained for the three values of laser power here analyzed (P=0.43, 0.61, and 0.67 W) are shown in Fig. 6b, where, for comparison, the speckle-free case (P = 0 W) is also shown.

Only in the absence of the optical speckle field, the MSD is always linear (slope equal to 1 in log–log scale) for the whole explored lag-time range, as expected for free-diffusion. In the presence of the optical speckle field, we observe a clear deviation from linearity. It is possible to identify three regions: a first region (I) where the MSDs are Fickian (slope = 1); a second region (II) characterized by sub-diffusive MSDs (slope < 1); a third region (III) in which MSDs return Fickian (slope = 1). This behavior can also be clearly appreciated from the plots of TAMSD/$\Delta t_n$ and MSD/$\Delta t_n$, as shown in Fig. 6c and d.

The physical origin of the observed trend of the MSDs is the interaction of microparticles with the optical potential. For short lag-time (region I) microparticles explore a region with MSD $<1 \,\upmu$m$^2$, i.e. smaller than the mean area of the grains ($\Delta _g^2\sim 16 \,\mu$m$^2$), and typically probe the quite uniform optical intensity characterizing the core of the speckle grain. Thus, their motion is not yet affected by the gradient force of the optical field. Increasing $\Delta t_n$, microparticles start exploring a wider region and, hence, feeling the optical force within the speckle grain (optical potential well) by experiencing a cage effect responsible for the sub-diffusive behavior of region II. In addition, the higher the laser intensity, the sooner this sub-diffusive trend begins (see Fig. 6b). At larger lag-times, particles escape their original speckle grain and start visiting other grains, leading to the restoration of linearity, which marks the beginning of region III.

### Identification of the spatio-temporal regions

To quantitatively identify the three regions introduced above, we start by considering that the trend of the MSD can be described, at least on a restricted time range, by a power law:

5 $$\begin{aligned} \text {MSD}\propto t^{\alpha } \end{aligned}$$

or, considering the logarithm of both members of (5), we have

6 $$\begin{aligned} \log (\text {MSD})=\alpha \log (t)+c \end{aligned}$$

Accordingly, $\alpha$ can be also defined as $d\log (MSD)/d\log (t)$ and corresponds to the local slope of the MSD in a log–log scale. Thus, the value of $\alpha$ can be used to estimate to what extent the MSD is consistent with linear diffusion ($\alpha$ = 1). Indeed, for the case of free-diffusion (P = 0 W), $\alpha =1$ over the entire range of $\Delta t$. Differently, in the presence of optical speckle field, the slope $\alpha$ changes with $\Delta t$, being always smaller than unity.

Observing that the experimental values of $\alpha$ for the speckle-free case fluctuate around 1 in a range of $3\%$ for long lag times and in a range of $1\%$ for short lag times, we assume these ranges as confidence intervals to establish a deviation from the linear case. Thus, the characteristic time $\tau _{\text {I}/\text {II}}$, which denotes the transition from region I to II, and $\tau _{\text {II}/\text {III}}$ which indicates the transition from region II to III, are estimated.

The estimated values $\tau _{\text {I}/\text {II}}$ and $\tau _{\text {II}/\text {III}}$ for each laser power, calculated following the above-described procedure, are listed in Table 1.

**Table 1:** Comparison between characteristics times ($\tau _{\text {I}/\text {II}}$ and $\tau _{\text {II}/\text {III}}$) and lengths ($\sqrt{\text {MSD}_{\text {I}/\text {II}}}$ and $\sqrt{\text {MSD}_{\text {II}/\text {III}}}$) at different optical powers.

P [W]	$\tau _{\text {I}/\text {II}} [s]$	$\sqrt{\text {MSD}_{\text {I}/\text {II}}}\ [\mu \text {m}]$	$\tau _{\text {II}/\text {III}} [s]$	$\sqrt{\text {MSD}_{\text {II}/\text {III}}}\ [\mu \text {m}]$
0.43	0.25	0.33	180	6.93
0.61	0.16	0.26	210	6.77
0.67	0.13	0.24	400	6.51

In the same table, we also report  the square root of the MSD, $\sqrt{\text {MSD}_{\text {I}/\text {II}}}$ and $\sqrt{\text {MSD}_{\text {II}/\text {III}}}$, obtained in correspondence of the time $\tau _{\text {I}/\text {II}}$ and $\tau _{\text {II}/\text {III}}$, as estimated from the curves of Fig. 6e and f.

The transition from region I to region II is reached at shorter times for higher laser power as expected from the increasing cage effect. On the other hand, the transition times from region II to region III are obtained on longer times for higher power when the root MSD is in the range $6.51 - 6.93\,\upmu \textrm{m}$, about 1.7 times than the grain size ($\Delta _g\sim 4\,\upmu$m).

### Displacements distributions

**Figure 7:**
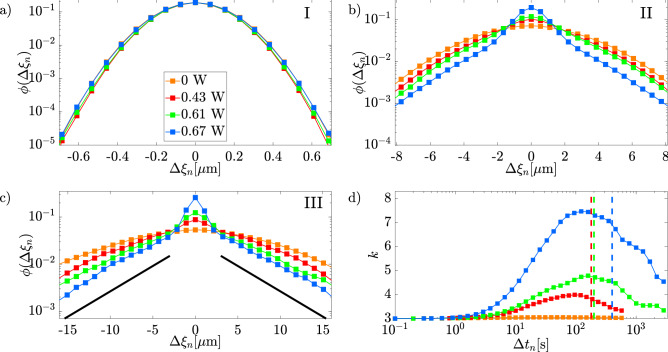
DDs in semi-logarithmic scale for (**a**) $\Delta t_n=0.1\,$s, (**b**) $\Delta t_n=50\,$s, and (**c**) $\Delta t_n=410\,$s at different optical powers. Black curves in panel (**c**) are guides to the eyes corresponding to exponential decay,  with a slope close to the one of the DD at P=0.67W (blue); Panel (**d**) represents the kurtosis *k* values of the DDs as a function of the lag time $\Delta t_n$.

We now analyze the DDs in the three regions I, II, and III. To do that, we evaluate the displacements of each trajectory as

7 $$\begin{aligned} \Delta \xi _{(k,n)}=\xi (t_k+\Delta t_n)-\xi (t_k) \ \ \ \forall k=1, \ldots ,N-n \end{aligned}$$

where $\xi =x,y$ while $\Delta t_n$ has a fixed value. By sampling the displacements over all the available trajectories and *k* values, we obtain the probability distributions $\phi _(\xi _n)$ at different values of *n*, i.e. corresponding to different lag-time. The probability distributions at different optical power are shown for three values of the lag-time $\Delta t_n=0.1,\,50,\,410\,\textrm{s}$, in Fig. 7a-c, respectively.

For region I, the DDs are well fitted by a Gaussian curve, confirming that, in the first region, Fickianity and Gaussianity are preserved. In region II (sub-diffusive motion), DDs start to deviate from the expected Gaussian shape, showing exponential tails that are fully developed for sufficiently large times^26,27^ (see Fig. 7b). When the motion returns Fickian (region III), the Gaussianity is not yet recovered and DDs still display exponential tails (see Fig. 7c) as expected for FnGD^26,27,48^. In Fig. 7b and c, it can be noted an over-statistics for small displacements, which can be ascribed to the optical confinement effect on the particles. In fact, increasing the power of the optical speckle field and, in turn, the particle confinement, the over-statistics of the central part becomes more visible.

To better represent how DDs deviate from gaussianity, it is useful to introduce the kurtosis evaluated as

8 $$\begin{aligned} k(\Delta t_n)=\frac{E[(\Delta \xi _{(k,n)}-\mu _n)^4]}{E[(\Delta \xi _{(k,n)}-\mu _n)^2]^2} \end{aligned}$$

where *E*[*x*] is computed over all available trajectories and k values for a given value of n, and $\mu _n=E[\Delta \xi _{(k,n)}]$. Plotting the values of the kurtosis for the case P$=0\,$W (see Fig. 7d), it is observed, that $k(\Delta t_n)$ is flat and always close to 3, as expected for a standard Brownian motion, which confirms that the DDs are Gaussian for the entire range of lag time analyzed. In the presence of the optical speckle field, however, the kurtosis is consistent with 3 at short lag-times only. At longer lag-times, the kurtosis increases, consistently with the non-Gaussian deviation observed in the DDs, reaching a maximum value, which increases on increasing the optical power, before the end of region II. The following decrease does not attain the value 3 within our observation time, although this condition is expected to occur in the long-time limit.

In the region III, the MSD is Fickian but the DDs do not yet follow a Gaussian distribution. It is reasonable to expect that, for sufficiently long observation times, the DDs tend towards Gaussianity as expected from theoretical arguments^22–24^. Due to the limited experimental time-window, the full return to Gaussianity can not be observed, although it can be foreseen from the trend of the experimental kurtosis of Fig. 7d. As a matter of fact, longer experiments are not easy to perform, as the possibility that a particle exits the experimental field of view (and is no more trackable) becomes more and more likely as time goes on. Thus, future longer experiments should be carefully designed to prevent or limit the impact of this issue.

## Numerical simulations

**Figure 8:**
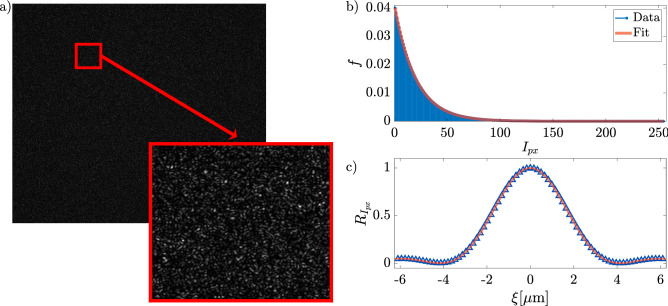
(**a**) simulated optical speckle field of $2000\,\upmu \text {m}\times 2000\,\upmu \text {m}$ and zoom of a region of $78\,\upmu \text {m}\times 78\,\upmu \text {m}$ (inset), (**b**) histogram of pixel intensity, and (**c**) auto-correlation function along x and y direction.

To elucidate the dynamics in our system, including the possible Gaussian recovery at large lag-times, we performed numerical simulations of Brownian point-like particles in an energy landscape mimicking the one induced by the optical speckle fields in experiments. It is hard to explicitly take into account the effective interaction felt by a finite-size particle when exploring a speckle field of comparable grain size, like in our experiments. Indeed, the resulting interaction on a particle is a complex superposition of the “local interaction” felt over the total volume occupied by the particle. Notably, the full three-dimensional nature of the adopted colloidal tracers makes the prediction of the effective interaction even more problematic. Hence, it is interesting to check to what extent experimental data can be captured by the simplified approach adopted in our simulations, namely by modeling the interaction of a particle with the speckle field as that of a simple point-like object placed in the particle center and using dipole approximation to calculate optical forces.

Indeed, after describing in detail our simulations, here we validate their outcomes through a direct comparison with experiments. In addition, we show how these simulations allow to cover a time-window larger than the experimental one, getting closer to the full recovery of Gaussianity.

Our simulation is based on the Langevin equation at low Reynolds numbers, in which the external force term is the optical force originated by the optical speckle field^49^, i.e.

9 $$\begin{aligned} {\left\{ \begin{array}{ll} \dot{x}=-\frac{1}{\gamma }F_x-\sqrt{\frac{k_B T}{\gamma }}\zeta _x \\ \dot{y}=-\frac{1}{\gamma }F_y-\sqrt{\frac{k_B T}{\gamma }}\zeta _y \end{array}\right. } \end{aligned}$$

where (*x*, *y*) are the coordinates of the microparticle center, $\gamma =3\pi \eta d$ is the friction coefficient, $\eta$ the viscosity of the surrounding medium, *d* the diameter of the microparticle, and $\vec {\zeta }=(\zeta _x,\zeta _y)$ is a white noise produced with a random generator of Gaussian distributed numbers. $\vec {F}=(F_x,F_y)$ is the optical force experienced by a point-like microparticle, given by the relations^50^

10 $$\begin{aligned} {\left\{ \begin{array}{ll} \vec {F}(\vec {r})=\frac{1}{4}\Re (\mathcal{A})\vec {\nabla }I(\vec {r})\\ \mathcal{A} =\frac{1}{2}\pi n^2_m\epsilon _0d^3\frac{n^2_p-n^2_m}{n^2_p+n^2_m} \end{array}\right. } \end{aligned}$$

being $n_p$ and $n_m$ the refractive index of the particle and the surrounding medium, $\mathcal{A}$ the polarizability given by the Clausius–Mossotti relation, and $\Re (\mathcal{A})$ its real part. In our experiment, the size of the particles is comparable to the laser wavelength; thus, to calculate the forces acting on the particles, the Generalized Lorenz–Mie theory should be used^46,47^. These calculations are very intensive and require knowledge of the optical field. Therefore, in our simulations, we instead use the Rayleigh approximation, which allows us to obtain trajectories that, as shown below, give a very good agreement with the experimental TAMSDs. Notice also that this approximation enables us to calculate the forces acting on the particles directly from the intensity of the optical speckle field.

In our simulations, the optical speckle field intensity $I(\vec {r})$ is simulated to get an arbitrarily large optical field. The algorithm for optical speckle field simulation can be summarized in the following steps:

- conversion of mask pixels intensity pattern *I*(*x*, *y*) into wave front phase values through the relation
  11 $$\begin{aligned} \Theta (x,y)=\frac{2\pi }{216} I(x,y) \end{aligned}$$
  that emulates the conversion pixel-phase of the SLM;
- generation of a phase-modified wave plane using the relation
  12 $$\begin{aligned} E_{sim}(t,\vec {r})=E_0\exp \left[ i\omega t-\vec {k}\cdot \vec {r}+\Theta \right] \end{aligned}$$
  that reproduces the action of the SLM on the experimental laser field with wave vector $\vec {k}$ and angular frequency $\omega$;
- generation of the optical field intensity in far field condition, $\Im _{sim}$, evaluating the square modulus of the bi-dimensional fast Fourier transform^51^ of equation (12).

Using the experimental calibration factor of the CCD, the simulated optical field has an extension of $2000\,\upmu \text {m}\times 2000\,\upmu \text {m}$, about ten times larger than the experimental one (Fig. 8a) and, to validate its goodness, we have verified that the pixel intensity distribution exhibits an exponential form and that the correlation function is well described by Eq. (2). Fig. 8b and Fig. 8c show the intensity distribution and the correlation function respectively, in a good agreement with the experimental behaviors.

**Figure 9:**
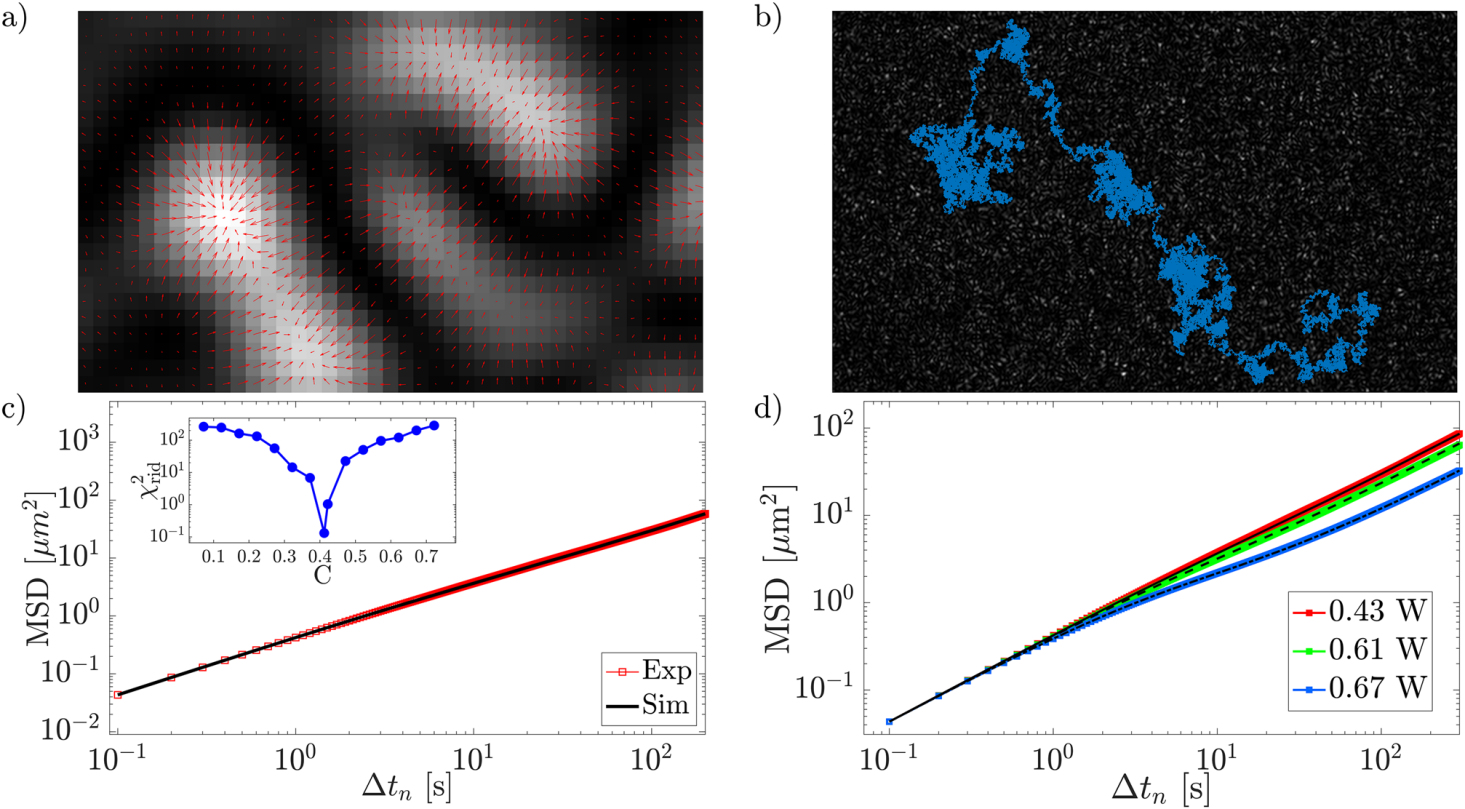
(**a**) force field (red arrows) of simulated optical speckle field around a few grains; (**b**) a typical simulated trajectory; (**c**) simulated and experimental MSD for the lowest optical power $P= 0.43\, W$, in the inset $\chi ^2_{rid}$ as a function of *C*; (**d**) simulated (black lines) and experimental (colored lines) for different optical powers, as indicated.

Using Eq. (10), the optical force produced by the simulated optical speckle field is

13 $$\begin{aligned} \vec {F}_{sim}(\vec {r})\equiv C\vec {\nabla }\Im _{sim} \end{aligned}$$

where *C* is the conversion factor force-numerical gradient that depends on the laser power. In order to estimate the values of *C* for the different experimental powers, we proceed as follows. We start with a test value of *C*, $C_{\text {init}}$, to obtain a preliminary force field through Eq. (13), see Fig. 9a. Once the optical forces are found, we calculate the particle trajectory using Eq. (9) for a random initial position of the particle in the optical speckle field (Fig. 9b). The trajectory is simulated in a time interval of $\text {T}=10^5\,\text {s}$ with a time step of $0.1\,\text {s}$. From the simulated trajectory, we find the mean square displacement. This procedure is repeated for 300 different particles and, finally, the averaged mean square displacement, MSD$_{sim}$, is obtained (Fig. 9c). From the values of MSD$_{\text {exp}}$ and MSD$_{\text {sim}}$ we evaluate the $\chi ^2_{rid}$ as

14 $$\begin{aligned} \chi ^2_{\textrm{rid}}(C)=\frac{1}{N-1}\sum _{n=0}^N\frac{\left[ MSD_{\text {exp}}(\Delta t_n)-MSD_{\text {sim}}(\Delta t_n,C)\right] ^2}{MSD_{\text {exp}}(\Delta t_n)} \end{aligned}$$

The best value of *C*, $C_{\textrm{opt}}$, which minimized the $\chi ^2$, is found for every experimental power. For $P=0.43\,\text {W}$, we find $C = 0.410$, see Fig. 9c, and for $P=0.61\,\text {W}$ and $P=0.67\,\text {W}$ we find $C = 0.474$ and $C = 0.615$, respectively. The effectiveness of our simulation is provided by the good agreement between $\textrm{MSD}_{\textrm{exp}}$ and $\textrm{MSD}_{\textrm{sim}}$ shown in Fig. 9d.

**Figure 10:**
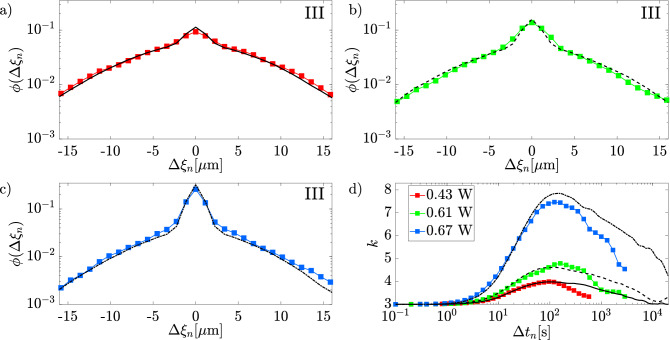
Comparison between the experimental (colored line) and the simulated (black line) DD at $\Delta t = 410\,$s at optical power $P= 0.43\,\text {W}$ (**a**), $P= 0.61\,\text {W}$ (**b**),and $P= 0.67\,\text {W}$ (**c**); (**d**) comparison between the experimental (colored lines) and the simulated kurtosis (black lines).

Once confirmed the agreement between simulated and experimental MSDs, which spans the entire experimental ranges of times and optical powers, we compute the DDs and the Kurtosis from simulation data. Fig. 10 demostrates that DDs from simulations reproduce quite well the experimental ones for all the laser powers in the early FnG regime (region III). Indeed, the comparison between the experimental and simulated kurtosis, shown in Fig. 10d, demonstrate a good agreement for time up to about the beginning of region III, where the curves start decreasing. Afterward, the spreading between experiments and simulations becomes evident: experimental curves start to decay much faster than the simulated ones toward the asymptotic value 3, which indicates the return to the Gaussianity.

This discrepancy can be likely ascribed to the point-like particle approximation used in our simulation. Indeed, a point-like particle explores a potential energy landscape that is proportional to the very local intensity gradient. Instead, the effective optical force acting on a finite-size particle arises from the superposition of intensity gradients distributed over the whole particle volume^42^, as mentioned above.

From the kurtosis of the simulations (black lines in Fig. 10d), it follows that the Gaussianity is reached roughly for $\Delta t\sim 10^4\,\text {s}$ for the lowest power case (solid black line in Fig. 10d). For higher powers, the return to Gaussianity is not observed within the simulated time-window, although, compared with experiments, the kurtosis attains values closer to the expected asymptotic value of 3. Overall, the difference between experiments and simulations suggests that the latter provide an overestimation of the time needed for the Gaussian recovery. Simulations can therefore be useful in the design of future experiments to provide an upper boundary to clearly observe a full return to Gaussianity.

## Conclusions

In this work, we described an experimental system of silica microparticles in water, where Fickian yet non Gaussian diffusion can be produced in a controlled and parametric way, by means of the forces generated by an optical speckle field. In particular, using a SLM, the optical speckle field can be easily tuned to change its properties (grain size, depth of the optical traps, etc). Investigating the MSD and the DDs, we observe three different regimes as the optical power increases: early free diffusion (Fickian and Gaussian) within the speckle grain in region I, subdiffusion at intermediate lag-times (non-Fickian and non-Gaussian) in region II, and FnGD in region III. We precisely identify these spatio-temporal regions through the characteristic times $\tau _{I/II}$ and $\tau _{II/III}$, and by the respective root MSDs, $\sqrt{MSD_{I/II}}$ and $\sqrt{MSD_{II/III}}$.

These time scales and the size of non-Gaussian deviations in the FnGD regime change with the optical power, which in fact controls the mean potential well height.

From the theoretical models^22–24^, we expect the presence of a fourth region where the motion is again purely Brownian, i.e. linear MSD and Gaussian DDs. However, we were not able to observe this region, due to experimental limitations.

We have complemented experimental results with Brownian dynamics simulations of point-like particles. Our simulations reproduce very well the MSD over the whole experimental time-window. As concerns the DD and the Kurtosis, the agreement with experiments is satisfactory up to time corresponding to the early FnGD, whereas some devitations emerge at larger times. In particular, the long-time Gaussian recovery seems to be slower in the simulation than in the experiment, which we ascribe to the adopted point-like approximation of the simulated particles. In spite of this, we are able to observe, within the simulated time-window, that the kurtosis generally gets closer than experiments to the expected Gaussian value of 3. For the simulation at the smallest optical power, we are also able to observe the full return to Gaussianity. Overall, the here presented simulations can provide a useful upper boundary for the duration of upcoming experiments targeted to monitor a full restoration of Gaussianity.

As a matter of fact, further and longer experiments on the here-described model-systems are on-demand to advance our understanding of the yet fully open issue of FnGD, and to validate recent theoretical models.

## Data Availability

Relevant data supporting the key findings of this study are available within the article. All data generated during the current study are available from the corresponding author upon reasonable request.
